# Function and regulation of transcription factors during mitosis-to-G1 transition

**DOI:** 10.1098/rsob.220062

**Published:** 2022-06-01

**Authors:** Mário A. F. Soares, Raquel A. Oliveira, Diogo S. Castro

**Affiliations:** ^1^ i3S Instituto de Investigação e Inovação em Saúde, IBMC Instituto de Biologia Molecular e Celular, Universidade do Porto, 4200-135 Porto, Portugal; ^2^ Instituto Gulbenkian de Ciência, 2780-156 Oeiras, Portugal

**Keywords:** transcription factor, mitotic bookmarking, mitosis-to-G1 transition, chromatin, electrostatic interactions, sequence-specific binding

## Abstract

During cell division, drastic cellular changes characteristic of mitosis result in the inactivation of the transcriptional machinery, and global downregulation of transcription. Sequence-specific transcription factors (TFs) have thus been considered mere bystanders, devoid of any regulatory function during mitosis. This view changed significantly in recent years, upon the conclusion that many TFs associate with condensed chromosomes during cell division, even occupying a fraction of their genomic target sites in mitotic chromatin. This finding was at the origin of the concept of mitotic bookmarking by TFs, proposed as a mechanism to propagate gene regulatory information across cell divisions, by facilitating the reactivation of specific bookmarked genes. While the underlying mechanisms and biological significance of this model remain elusive, recent developments in this fast-moving field have cast new light into TF activity during mitosis, beyond a bookmarking role. Here, we start by reviewing the most recent findings on the complex nature of TF–chromatin interactions during mitosis, and on mechanisms that may regulate them. Next, and in light of recent reports describing how transcription is reinitiated in temporally distinct waves during mitosis-to-G1 transition, we explore how TFs may contribute to defining this hierarchical gene expression process. Finally, we discuss how TF activity during mitotic exit may impact the acquisition of cell identity upon cell division, and propose a model that integrates dynamic changes in TF–chromatin interactions during this cell-cycle period, with the execution of cell-fate decisions.

## Graphical abstract

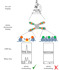

## 1. Introduction

Sequence-specific transcription factors (TFs) are key components of gene expression in virtually every biological process. Their activity is thus tightly controlled by a wide-variety of mechanisms. Binding of a TF to a gene regulatory region is to large extent dependent on its concentration [1]. Eukaryotic cells have therefore developed a series of mechanisms to modulate the concentration of TFs, and ultimately their function. At a broader level, this regulation may be achieved by tampering with the balance of TF gene transcription, translation and protein degradation. Additional mechanisms impact concentration at subcellular compartments, for example via the regulation of TF nuclear translocation, or even nucleocytoplasmic shuttling. In many cases, dimerization with regulatory partners can promote or inhibit TF function, determining the concentration of functional TF [2]. More recently, the inclusion of TFs in liquid–liquid phase separated (LLPS) condensates emerged as a novel mechanism to regulate their concentration near chromatin [3].

Mitosis is characterized by drastic cellular changes that altogether result in a global downregulation of transcription [4]. In addition to impacting the basal transcriptional machinery, mitosis affects the function of TFs at various levels. Nuclear envelope break-down (NEB) results in a sudden dilution of nuclear proteins, which mathematical modelling predicts negatively impacts TF activity [5]. Moreover, post-translational modifications (PTMs) that occur exclusively during mitosis have been documented to influence TF activity [6,7]. Adding to this, chromatin becomes highly condensed and modified, hampering the activity of TFs and other regulatory proteins, with many disengaging from mitotic chromosomes [4].

In spite of the above, the finding that an increasing number of TFs is able to associate with mitotic chromosomes, sometimes even occupying part of their cognate binding sites in mitotic chromatin, is driving a paradigm change [6,8]. Although the nature and function of such interactions are still poorly understood, they suggest TFs may play important roles in a continuous flow of regulatory information across cell divisions, thereby impacting the acquisition of cell identity by newborn cells. In addition, other recent studies suggest that at least in some cell types, gene expression restarts earlier than previously appreciated, in transcriptional waves during mitosis-to-G1 (M-G1) transition [9–12]. Altogether, these findings urge the need to reexamine the role TFs may play, orchestrating a complex cascade of events that unfold during this time window. Here, we discuss the most recent literature addressing the nature and regulation of interactions of TFs with chromatin during M-G1, and how this may ultimately impact the acquisition of cell identity.

## 2. Distinct forces mediate binding of TFs to chromatin

Distinct types of interactions mediate the association of TFs with DNA. Long-lasting sequence-specific interactions are established between residues in the DNA-binding domain (DBD) and DNA bases within consensus sequence recognition motifs. In addition, TFs engage in non-specific interactions with chromatin, mediated primarily by electrostatic forces that do not rely on specific DNA sequences [13–15]. These interactions are thought to facilitate the search for sequence-specific sites. While certain TFs encounter their target genes using mainly three-dimensional (3D) diffusion, others also engage in electrostatic one-dimensional (1D) sliding along the DNA, while scanning the genome. This often relies on the DBD, with the same residues switching role from a purely electrostatic interaction with the DNA backbone, to a highly specific binding mode [1,16]. Thus, a correct balance between these two modes of binding is required for maximum efficiency in the search for target regions in the genome [1,17].

While it was initially thought that complexation with positively charged histones neutralized the negative charges of DNA molecules, both computational and experimental work concluded nucleosomal DNA has similar net charges to naked DNA [18,19]. In spite of decreased global histone acetylation levels during mitosis, it is at this stage that the negative electrostatic field of DNA is strongest, as result of high chromatin compaction and H3ser10 phosphorylation throughout chromosomes [4]. Thus, electrostatic interactions between proteins and DNA play a more prominent role in the context of condensed chromosomes during mitosis, or in DNA-dense and heterochromatic regions in the interphase nucleus [8,20]. Hence, binding of TFs to mitotic chromosomes should be discussed considering the different types of interactions that TFs may engage on mitotic chromatin.

## 3. Electrostatic interactions and mitotic chromosome binding

Live-cell imaging experiments using fluorescently tagged proteins have been a major drive in recent studies characterizing how TFs interact with mitotic chromosomes (box 1). Using this approach, a growing number of TFs has been shown to colocalize with condensed chromosomes during mitosis, a property herein referred to as ‘mitotic chromosome binding’ (MCB) [8,20,27–29]. Despite the growing number of TFs reported to display MCB, it remains less clear how this binding is mediated. Site-directed mutagenesis experiments highlighted the role of non-specific interactions in MCB. In a study of FoxA1, a combined mutation of two residues that contact the DNA back-bone (affecting only mildly sequence-specific binding in *in vitro* assays), dramatically diminished binding to mitotic chromosomes [30]. In a recent study on Brn2 (also termed POU3f2), a point-mutation in a residue predicted to interact with the DNA back-bone (but not DNA bases), abolishes MCB in neural stem cells. Conversely, a phospho-mimetic Brn2 derivative devoid of sequence-specific binding (S362D), retains to large extent its ability to interact with mitotic chromosomes [20]. On a different scale, a large screening based on live-cell imaging analysis of TFs ectopically expressed in embryonic stem (ES) cells, concluded that approximately 20% out of 500 mouse TFs show MCB ability (albeit to various degrees). Importantly, a significant correlation was found, between MCB, and the TF electrostatic properties, in particular of its DNA-binding domain [8].

Box 1. **The confounding effects of chemical fixation.**

Understanding the impact of chemical fixation when assessing TF–chromatin interactions has helped to reconcile recent observations with old data. Formaldehyde fixation is widely used both in immunofluorescence and Chip-seq protocols. This fixator was found to artifactually exclude most TFs from mitotic chromosomes as observed by immuno-staining [21,22], raising the hypothesis that it could be preferentially fixing strong sequence-specific binding instead of more transient interactions with chromatin. Indeed, the general TF TBP, which retains binding to most of its interphase targets during mitosis, can be fixed using formaldehyde and still be enriched at mitotic chromosomes as seen by immunocytochemistry [21,23]. In contrast, the TF ESRR*β*, when fixed and visualized with the same protocol, cannot be observed at mitotic chromosomes, although significant mitotic binding can still be detected using ChIP-seq [24]. Interestingly, using fixators that crosslink protein-protein interactions (e.g. DSG or glyoxal), the MCB of ESRR*β* and Sox2 is preserved whereas Oct4 is not [24], in line with other studies showing Oct4 low MCB activity [25] and low non-specific affinity to chromatin *in vitro* [26]. Thus, in light of current knowledge, initial immunolabelling studies in fixed cells, suggesting the eviction of certain TFs from mitotic chromatin, may have to be revisited using live-cell imaging analysis.

The transient nature of interactions underlying MCB was revealed by studies using fluorescent recovery after photobleaching (FRAP) and single molecule tracking (SMT), showing decreased TF residence time on mitotic chromatin as compared to interphase [21,25,30,31]. Along the same line, fluorescent loss in photobleaching (FLIP) experiments showed TFs are constantly disengaging from mitotic chromosomes, travelling long distances before associating again with chromatin. Altogether, these experiments resulted in an important paradigm shift, whereby TFs associate with mitotic chromosomes in a highly dynamic process, as opposed to remaining trapped upon chromatin condensation, as originally thought [24].

## 4. Mitotic bookmarking versus mitotic chromosome binding

While growing evidence indicates MCB observed by live-cell imaging analysis reveals mostly electrostatic interactions, in some cases this phenomenon occurs concomitantly with sequence-specific binding. This is difficult to demonstrate using mutagenesis, given that mutations affecting sequence-specific binding may also disrupt electrostatic interactions [1,16]. The characterization of sequence-specific binding by TFs can instead be carried out by chromatin-immunoprecipitation followed by sequencing (ChIP-seq), using chromatin extracted from synchronized cell populations. Following this approach, several TFs were found to bind in a sequence-specific manner to regulatory regions in mitotic (prometaphase) chromatin [29,31,32]. This has been proposed to mark a subset of genes for efficient reactivation upon mitotic exit, resulting in the concept of mitotic bookmarking as a mechanism of gene regulatory memory in dividing cells. The gene bookmarking model foresees that mitotic chromatin is more accessible than previously assumed. Indeed, at the macromolecular level, DNase-seq, ATAC-seq and MNAse-seq revealed a global maintenance of chromatin accessibility in mitosis when compared to interphase [21,24,33]. Nevertheless, using ES cells, MNAse-seq allowed to visualize in more detail the fine differences in mitotic chromatin organization, showing that nucleosome positioning is maintained specifically at ESRR*β* and CTCF bookmarked sites, counteracting large chromatin rearrangements characteristic of this stage [24,34]. This observation provides a mechanistic framework for mitotic bookmarking by TFs, although the functional impact on gene expression remains to be properly addressed.

**Figure 1.**
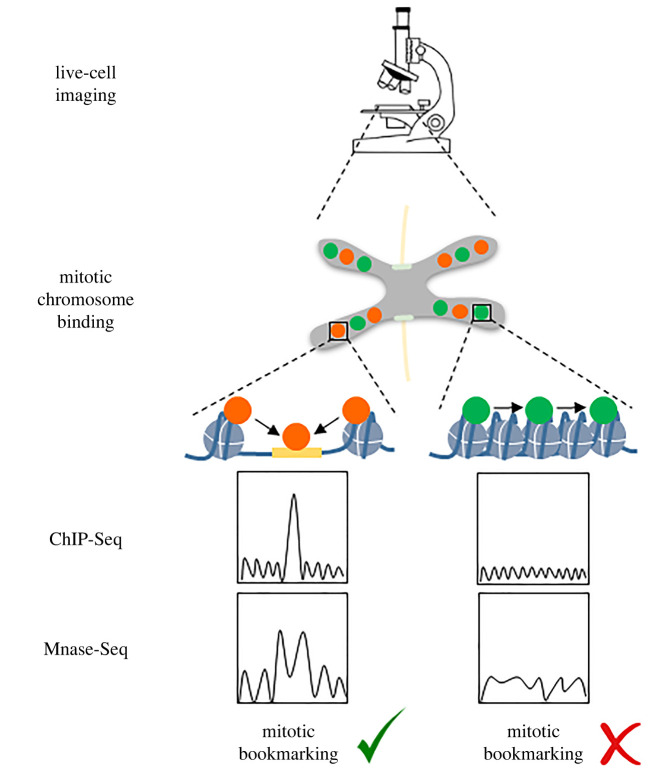
Mitotic chromosome binding (MCB) is not synonymous with mitotic bookmarking*.* The colocalization of TFs with mitotic chromatin as observed by live-cell imaging microscopy (here referred to as mitotic chromosome binding, MCB) is primarily the result of electrostatic interactions, and does not necessarily entail sequence-specific binding. Sequence-specific binding, a prerequisite for mitotic bookmarking activity, implies a direct binding of the TF to its target sequence and hence should be observable by ChIP-seq. Binding of a mitotic bookmarker TF has been shown to maintain nucleosome positioning in mitotic chromatin, which can be revealed by MNase-seq, but is not evident by ATAC-seq (see main text for references).

Altogether, available studies suggest that non-specific electrostatic interactions are the main determinants of the widespread binding of TFs in mitosis observed in live-cell imaging, and that in some cases such interactions contribute to sequence-specific binding to mitotic chromatin. As a consequence, only a subset of TFs that associate with mitotic chromosomes in live-cell imaging, are expected to display mitotic bookmarking activity as this was originally described (figure 1). In line with this prediction, in ES cells the two pluripotency TFs Sox2 and Oct4 have been reported to display MCB in the absence of sequence-specific binding, although contradictory ChIP-seq results in the literature await further clarification [24,32]. Nevertheless, nucleosome positioning is not maintained in mitotic chromatin at Sox2 and Oct4 target sites, clearly setting these TFs apart from the mitotic bookmarkers ESRR*β* and CTCF [24,34].

One possibility is that TFs that interact with mitotic chromosomes solely via electrostatic interactions may reside at the chromosome periphery, a domain covering the outer surface of mitotic chromosomes, which is formed by the accumulation of proteins and RNAs originated from the nucleolus [35,36]. Although imaging studies have excluded this possibility for Sox2 and Oct4, the overall contribution of this compartment to MCB by TFs remains to be properly addressed [24]. Ki-67 protein has emerged as an important organizer of the peri-chromosomal layer [35,36]. According to the current model, Ki-67 acts as a surfactant-like placing its positively charged N-terminal domain towards the outside of the chromosome periphery compartment [37]. In such a model, positively charged TFs could be repelled by this positively charged domain. Nevertheless, some TFs can overcome the Ki-67 barrier to bind negatively charged chromatin. What allows passage through this electrostatic barrier remains poorly explored. Understanding if the formation of LLPS condensates via intrinsically disordered regions (IDRs) of TFs can impact their access through the Ki-67 barrier should be addressed in future studies.

## 5. Mitotic chromosome binding: an intrinsic or regulated property of TFs?

The different behaviour of TFs with regard to the way they interact with mitotic chromatin (no binding, electrostatic-mediated binding or sequence-specific binding) raises a still controversial question in the field: is MCB an intrinsic property imposed by the biochemical characteristics of each individual TF, or is this an activity modulated by specific regulatory mechanisms? Below, we first review what is known of the structural determinants required for MCB, before discussing two possible major layers of regulation.

### 5.1. Structural determinants of MCB

Studies on MCB have initially associated this property with the ability of TFs to bind nucleosomal DNA (i.e. pioneer TF activity), a property known to rely on specific features of the DBD (box 2). Although recent studies have shown these are two distinct activities of TFs, the DBD does seem to play a central role in mediating association of TFs with mitotic chromatin. Accordingly, various studies have shown the DBD to be both required and sufficient for MCB [20,25,31]. Importantly, distinct classes of TFs (bearing distinct types of DBDs) display MCB, suggesting the absence of a common underlying mechanism. Interestingly, a systematic analysis of biochemical properties of TFs has found the most predictive feature of MCB to be the absolute charge per DBD, in line with the importance of electrostatic forces in this context [8]. Other protein domains may modulate MCB by the DBD. For example, in the case of NFAT5, a TF responsible for osmoprotective gene regulation, MCB binding is only observed upon truncation of its C-terminal domain [43]. This finding implies that the DBD has an intrinsic ability for MCB yet, other domains are able to hinder this property. If so, this opens the possibility that conformational changes that expose/hide the DBD may regulate MCB by TFs.

Box 2. **Mitotic chromosome binding and pioneer activity.**

The TF FoxA1 is perhaps the most paradigmatic mitotic bookmarker. This master regulator of liver development can bind its target sites on ‘closed chromatin’ (i.e. nucleosomal DNA), a property referred to as ‘pioneer activity’. This precedent linked mitotic bookmarking (and to some extent MCB) with pioneer activity, a rather intuitive association whose molecular basis however, failed to materialize. Indeed, sequence-specific TFs without pioneer activity have been reported to bind mitotic chromosomes, one example being Brn2 [20,38,39]. Conversely, examples of pioneer TFs that do not display MCB (at least in tested conditions), have started to emerge. Ascl1 is a well-characterized pioneer TF with a master regulatory function in neurogenesis, and which was recently shown to be excluded from mitotic chromatin in neural stem cells [20,40,41]. Another example is the pioneer TF Zelda, shown to be excluded from mitotic chromatin during zygotic genome activation in *D. melanogaster* [42]. Further supporting these observations, no correlation was found between the MCB of TFs ectopically expressed in ES cells and their ability to occupy nucleosomal regions [8]. Recent structural work on various pioneer TFs indicates pioneer activity does not result from a common DNA binding strategy [26]. As a consequence, the pioneer activity of distinct TFs may translate differently into MCB and mitotic bookmarking abilities.

**Figure 2.**
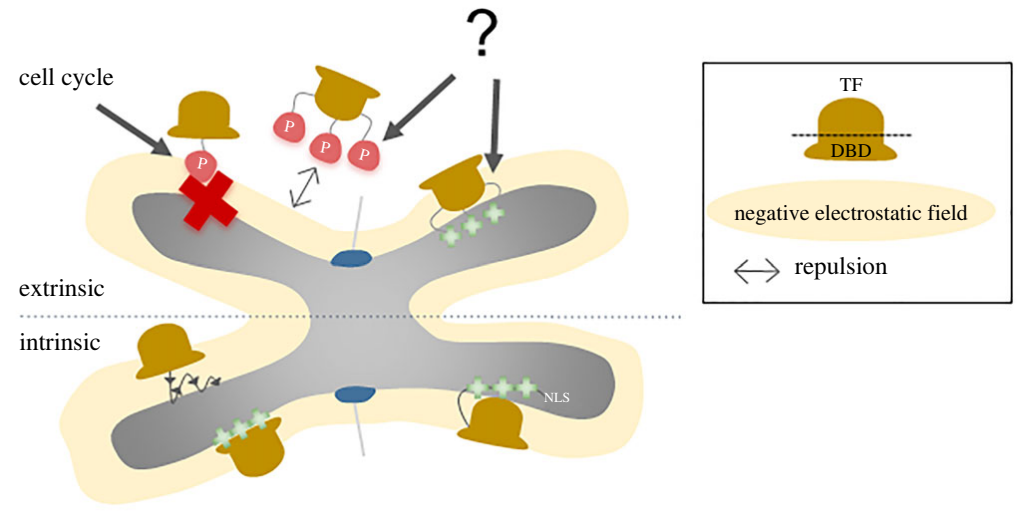
Intrinsic and extrinsic regulation of mitotic chromosome binding (MCB). The electrostatic potential of a TF, and in particular of its DBD, has emerged as a critical determinant of MCB. More positively charged DBDs will naturally bind and scan the mitotic chromosomes more efficiently than their less positive counterparts (bottom left). Nuclear localization signals (NLS) have also been shown to contribute to the binding of TFs to mitotic chromosomes due to their abundance in positively charged residues (bottom right). Having a strong intrinsic component, protein electrostatic properties can potentially be modulated by PTMs, this way providing an extrinsic mode of regulation of MCB (upper half). While this remains to be fully explored in future studies, existing evidence indicates mitotic-specific phosphorylation of residues within the DBD by cell-cycle regulators can hinder MCB (top left) (see main text for references).

In addition to the DBD, several reports described the ability of a nuclear localization signal (NLS) to promote the association of proteins with mitotic chromosomes. The initial interpretation of these findings came with some controversy, as they have been seen as evidence for a role of the nuclear import machinery driving MCB (box 3). The protein sequence of many NLSs is highly enriched in positive residues [46]. Thus, a rather simple explanation is that NLSs establish strong electrostatic interactions with highly condensed chromatin, promoting the association of proteins with mitotic chromosomes (figure 2). This is in line with a recent study, which found short sequences of either lysines or arginines to slide efficiently along DNA, functioning as so-called ‘molecular sleds’. Strikingly, too many positively charged residues decrease sliding speed, indicating the importance of properly balancing the strength of protein-DNA interactions [47,48]. In agreement with a non-canonical model of NLS function, introducing two alanines in the SV40 NLS sequence disrupts nuclear import, while maintaining the ability to promote binding to mitotic chromosomes of an YPet-NLS fusion. Conversely, a peptide composed of five positively charged residues is sufficient to promote or increase MCB of certain TFs [8]. In some cases, however, MCB is only achieved upon the addition of multiple NLSs, suggesting the need to counteract a much lower electrostatic potential. One example is Ascl1, which displays MCB only when fused with a tandem of six SV40 NLSs [20]. Regardless of the mechanistic basis, to which extent some of these experiments underline a role for the NLS in the context of a native protein is unclear, in particular when tandems of several NLS sequences were used. Moreover, not all NLS sequences promote mitotic chromosome binding in live-cell imaging studies [20,21]. Future studies should better characterize to which extent specific NLS sequences are predictive of mitotic chromosome binding, and whether other types of protein sequences can play a similar role.

Box 3. **Regulation by the nuclear import machinery?**

One of the first mechanisms proposed to regulate the association of TFs with mitotic chromosomes involved the nuclear import machinery, more specifically the Ran-GTP gradient which in interphase regulates nuclear import of proteins containing an NLS. Retention of the Ran-GTP promoting factor RCC1 on condensed chromosomes results in a gradient of Ran-GTP being maintained throughout mitosis, which is required for the activation and loading of Spindle Assembly Factors (SAFs) once these are at vicinity of mitotic chromosomes. It has been proposed that the Ran-GTP gradient could function as a ‘molecular GPS’ to direct specific sets of proteins such as SAFs to condensed chromosomes, a model that could be extended to other nuclear proteins such as TFs [44]. In support of this idea, live-cell imaging experiments revealed that retention of GFP (or a DNA-binding deficient Oct4) in the vicinity of mitotic chromosomes can be promoted by fusion to a NLS [21,25]. Moreover, a β-importin inhibitor (importazole) decreases the ability of a temperature-sensitive HNF1*β* mutant to interact with mitotic chromosomes upon cold shock, further implicating an NLS-based mechanism [45]. Although appealing at first, the molecular GPS model is difficult to conciliate with the current knowledge of how the Ran-GTP gradient promotes the activation of SAFs at the vicinity of chromosomes, a process which relies on the random diffusion of molecules that will eventually encounter high Ran-GTP concentration. Thus, evidence for a role of the Ran-GTP gradient in actively guiding proteins (including TFs) towards the mitotic chromosomes, is still lacking.

### 5.2. Importance of the chromatin landscape

The chromatin environment may provide one important level of regulation of MCB. For example, PTMs of core histones may influence the ability of a TF to bind mitotic chromatin by altering chromatin compaction, and/or the associated electrostatic field. A recent study tested the idea that repressive chromatin may hinder the association of TFs with mitotic chromosomes, by abolishing the mitotic levels of H3K9me3 in ES cells [49]. Somehow unexpectedly, depletion of this histone PTM leads to increased mitotic chromosome compaction, likely as result of concomitant increase of H3K27me3 and H3ser10ph. Importantly, retention of several TFs including the mitotic bookmarker ESSR*β* in such compacted chromosomes was reduced, as found by proteomics and immunofluorescence. Of note, no differences were observed at TF binding sites with ATAC-seq (investigated in case of ESRR*β*), thus not supporting changes of chromatin accessibility as the underlying mechanism of decreased TF retention [49]. Moreover, the MCB of TFs has been correlated with their colocalization at heterochromatic loci enriched for H3K9me3 in interphase [8,20]. Altogether, these observations indicate the importance of addressing how specific histone PTMs (and in particular H3K9me3) may impact the association of TFs with mitotic chromatin, and how this may result from their contribution to the electrostatic field.

Other players involved in chromatin condensation that may also regulate MCB are divalent cations, particularly Ca2+ and Mg2+. These are sufficient to induce chromatin condensation, either directly via electrostatic neutralization or indirectly by promoting the activity of non-histone proteins such as TopoII [50–52]. A transient rise in Mg2+ levels can be observed in live cells during mitosis, probably as a consequence of high ATP hydrolysis [53]. Although overall mitotic chromatin is negatively charged, such cations may promote the local increase of chromatin charge, reducing or canceling the negative electrostatic field [19,54]. It is therefore tempting to speculate that direct competition with divalent cations may define a mechanistic level, at which MCB and mitotic bookmarking by TFs may be regulated.

### 5.3. Targeting of TFs by PTMs

One way the cellular context can influence TF interactions with DNA is via their targeting by PTMs. In line with the pivotal role of phosphorylation in cell-cycle regulation, high-resolution mass spectrometry-based proteomics has shown around 70% of proteins are phosphorylated along the cell-cycle, with nuclear proteins at the top of the list [55]. Indeed, many TFs and other transcriptional players such as chromatin regulators are phosphorylated specifically during mitosis, usually resulting in their functional inactivation by various mechanisms. In case of TFs these include targeted degradation, or inhibition of sequence-specific binding (as assessed by *in vitro* assays) [6]. Importantly, TF phosphorylation can at least in some cases dissociate sequence-specific from non-specific binding, helping to explain why not all MCB TFs display *bona fide* mitotic bookmarking activity. A striking example is that of Brn2, with a phospho-mimetic form devoid of sequence-specific binding (S362D) retaining to large extent its MCB ability in neural stem cells [20]. In the case of Oct4, mitotic-specific phosphorylation by Aurora kinase B inhibits sequence-specific binding to target genes in ES cells, as assessed by ChIP-PCR. In line with this, a specific inhibitor of this kinase results in increased Oct4 binding at chromosome arms, although possible indirect effects of this inhibition on chromatin status, including histone de-phosphorylation, may also contribute to this observation [24,56]. Also in ES cells, live-cell imaging analysis has shown a phospho-mimetic version of CTCF at S224 to localize only to pericentric regions during metaphase, as opposed to non-phosphorylated CTCF found at chromosome arms. Surprisingly, this does not impact CTCF role in maintaining chromatin architecture, suggesting this phosphorylation to impact only transcriptional regulation [57].

Altogether, growing evidence suggests phosphorylation plays a role in modulating how TFs interact with chromatin during mitosis, although we have only begun to fully explore its functional importance in this context. Future studies should address whether mitotic phosphorylation (for example under the direct control of cell-cycle regulators) may explain the reported exclusion of some TFs from mitotic chromosomes, and what are the mechanisms that revert this inactivation process upon mitotic exit (figure 2).

Another important possibility is that cell-type specific phosphorylation events may confer distinct MCB abilities to one TF across different cell types. One illustrative example is that of CTCF, which in ES cells was shown using ChIP-seq to function as a mitotic bookmarker and maintain nucleosome positioning [34]. Bookmarking ability was also observed in erythroblasts using ChIP-seq, demonstrating it is not a unique characteristic of ES cells [58]. CTCF also associates with mitotic chromosomes in HeLa cells, NIH3T3 and C2C12 cells, as assessed by live-cell imaging or chromatin fractionation approaches [32,59,60]. However, CTCF does not act as a mitotic bookmarker in NIH3T3 and C2C12 cells, as assessed by ChIP-seq [34]. Furthermore, CTCF is excluded from prometaphase chromosomes in U2OS cells, as revealed by live-cell imaging and genomics approaches in two independent studies [11,61]. The indication that MCB can be dynamically regulated, suggests the presence of TFs on mitotic chromatin may hold functional relevance. Next, we discuss potential functions for MCB.

## 6. TF activity and waves of transcriptional reactivation during M-G1

Although the function of MCB and mitotic bookmarking of TFs remains largely unknown, one interesting possibility is that such interactions contribute to a temporal gradient of gene reactivation during mitotic exit. Initial studies had already proposed that sequence-specific binding to regulatory regions may result in early reactivation of bookmarked genes upon mitotic exit [29,30]. However, these studies lacked the temporal resolution required to survey transcription during mitotic exit. Instead, they mostly relied on correlative analysis, associating TF occupancy in mitosis with kinetics of reactivation of a strict number of transcripts during early G1. Stronger evidence was shown for ESRR*β*, where sequence-specific binding during prometaphase is predictive of earlier reactivation on a genome-wide scale [31]. The link between MCB and timing of gene reactivation has recently gained traction, as the latter process has been proposed to occur in a hierarchical fashion, in sequential waves throughout M-G1. This hierarchical model originates from studies using EU-seq metabolic labelling to profile transcripts with greater sensitivity and suggests a significant number of genes have their maximum transcriptional rate (defined as a ‘spike’) during mitotic exit [9,11,62]. Importantly, waves of gene transcription throughout M-G1 were confirmed in various cell types. These include HUH7 hepatoma, osteosarcoma and retinal pigment epithelial cells, and concluded that transcriptional ‘spiking’ during anaphase/telophase is a rather common event [11]. So far, the underlying significance of the temporal pattern observed remains poorly understood. In HUH7 hepatoma cells, the earliest transcriptional wave is enriched for genes with predicted function in growth and rebuilding of daughter cells (i.e. housekeeping) [9]. By contrast, the timing of transcriptional reactivation of house-keeping and pluripotency genes does not differ significantly in ES cells, which are characterized by an overall faster kinetics of gene reactivation [12,63]. This may result from constraints imposed by the cell-cycle in ES cells, namely their shorter G1 phase. Understanding what can be the biological significance of hierarchical gene reactivation in each cellular context is a pressing question to be addressed in future studies.

Other studies on three-dimensional chromatin architecture provided further support to the hierarchical model of gene reactivation. In mitotically arrested erythroblasts, the reestablishment of enhancer-promoter contacts starts shortly after nocodazole release, reaching maximum contact in early G1 [64]. Formation of enhancer-promoter contacts occurs concomitantly with enhancer usage, as evidenced by the progressive appearance of enhancer RNAs (eRNAs) during M-G1 [9,10]. Subsequent studies addressed how other higher-order interactions are reorganized into functional domains, to potentiate proper gene regulation. In erythroblast cells, structural loops are formed by CTCF/cohesin complex gradually throughout mitotic exit, with the appearance of topologically associated domains (TADs) starting as early as anaphase/telophase. Importantly, more than half of enhancer-promoter contacts occur independently of CTCF/cohesin and display strong interactions in anaphase/telophase, being more rapidly formed and in larger numbers than structural loops during this period [58]. In HeLa cells, the majority of enhancer-promoter contacts were identified in telophase/cytokinesis, although also before structural loops are reformed [58,65]. Interestingly, mitotic-specific CTCF degradation has a marginal impact on gene reactivation during M-G1 in both ES and erythroblasts, in line with a preponderant role for enhancer-promoter contacts in driving gene expression prior to the establishment of structural loops [63,66].

Altogether, accumulated evidence shows gene reactivation during M-G1 results from a highly coordinated succession of events, and suggests TFs may play an important regulatory role. This idea is supported by a recent study, which used single-molecule RNA-FISH (smRNA-FISH) as a method to image unprocessed transcripts at places of active transcription in single cells, in this way probing with high temporal resolution and sensitivity, the reactivation of specific transcripts in neural stem cells [20]. In line with a hierarchical model, the onset of reactivation of the NS cell gene Nestin was observed in anaphase, as opposed to the gene encoding Notch ligand Dll1, only starting to be transcribed in early G1. Importantly, the use of a mitotic-specific dominant negative form of Brn2 concluded that its canonical TF activity is required for early reactivation of Nestin during M-G1. However, Brn2 MCB was shown to rely on electrostatic interactions rather than sequence-specific binding to prometaphase chromosomes. These findings rule-out that early Nestin gene reactivation results from a *bona fide* mitotic bookmarking activity of Brn2. Instead, this study proposed an alternative model, whereby increased TF concentration on chromatin (promoted by Brn2 MCB) fosters the early reactivation of its target transcripts. In line with this possibility, the late reactivation of Dll1 observed in neural stem cells correlates with increased nuclear concentration (upon nuclear-envelope reformation) of its main activator Ascl1, which remains excluded from mitotic chromatin during M-G1 [20]. However, support for this model is somehow limited, given the reduced number of transcripts analysed.

With the aim of providing a large scale understanding of a regulatory role of TFs during M-G1, a recent study used mathematical modelling to integrate transcriptional profiling of gene reactivation, with *in silico* predictions of binding sites, and MCB data from a large number of TFs [8,9,67] Although a simple correlation between MCB and the timing of reactivation was not found (with FoxA1 being among the least active TFs during mitosis) this study did identify distinct groups of TFs that display maximum activity at different time points during M-G1, in line with the existence of a TF hierarchy during mitotic exit. One important limitation of this work however, is that it only considered TF binding at proximal promoter regions, ignoring the important role enhancers play in tissue-specific gene regulation. Moreover, gene regulation is often the result of combinatorial action of multiple TFs, and it is most likely that additional determinants to MCB will need to be considered if one is to decipher the temporal logic of gene reactivation during M-G1.

Understanding the role of TFs during mitotic exit will most likely require an integrative view of TFs and their cofactors during this time window, several of which have been shown to interact with mitotic chromatin [6]. Given its ubiquitous role in mediating TF gene activation, one important example is CBP/p300, which catalyses H3K27ac [68–70]. Interestingly, H3K27ac is reduced during prometaphase, reappearing in anaphase/telophase with a pattern that mirrors interphase occupancy [11,71]. Such changes over time suggest a process of re-deposition that is unlikely to rely on a self-propagating chromatin mechanism. Importantly, reducing H3K27ac with a new selective inhibitor of the catalytic subunit of CBP/p300 impairs gene reactivation during mitotic exit (even if inhibition only takes place in anaphase/telophase), indicating H3K27ac recovery during this time period is important for reactivation [11,12]. Interestingly, depleting mitotic RNA pol II leads to reduced H3K27ac levels and cohesin loading in early G1, hinting at a possible link between canonical TF activity and H3K27ac deposition during M-G1 [72]. Altogether, these observations also cement the importance M-G1 time-window may have for cell identity maintenance and cell fate changes.

## 7. Temporal pattern of transcriptional reactivation and cell fate

The large-scale changes in genome and chromatin organization characteristic of mitosis, associated with major downregulation of transcription [9], are thought to make this cell-cycle stage the perfect platform for cell fate transitions [73,74]. Several studies have explored this topic using ES cells as a model system. In one report, neuroectodermal commitment of human ES cells was shown to occur only during late G1, by regulation imposed through G1 CDKs on TGF-β signalling response [75]. By contrast, endoderm/mesoderm fate commitment takes place solely within a narrow time-window in early G1, while G1 CDKs are not still fully active, suggesting differentiation during this period may be connected with earlier events in M-G1 [75,76]. How the activity of TFs during M-G1 impacts the identity of daughter cells is still poorly understood. Targeting Sox2 and Oct4 for degradation using a cyclin B1 mitotic degron (which results in protein degradation from anaphase to 1 h into G1), confirmed their importance during this period for robust pluripotency maintenance [25,32]. Sox2 degradation during M-G1 transition completely failed to promote neuroectodermal commitment, in agreement with the idea that a small increase in Sox2 levels during G1 is required to bias ES cells toward the neuroectodermal lineage [77]. Interestingly, Sox2 overexpression during other stages of the cell cycle was not sufficient to compensate for the lack of Sox2 during M-G1. Nevertheless, Sox2 and Oct4 roles during M-G1 may be differently important, as only Oct4 presence during this period is required for efficient reprogramming of fibroblasts into induced-pluripotent stem (iPS) cells [25,32].

Recently, an elegant study performed in ES cells characterized the genome-wide binding of Sox2/Oct4 along the cell-cycle, and how this correlates with chromatin accessibility, providing important mechanistic information. Here the authors report that recovering Oct4 levels after its short and acute depletion during M-G1 transition, results in some Oct4-dependent genomic regions becoming permanently inaccessible [78]. Surprisingly, Sox2 can maintain the accessibility of some genomic sites co-occupied with Oct4 but not others, suggesting the existence of a hierarchy of genomic regions, defined by their accessibility [78]. This hierarchical model may also explain the different requirements observed for Sox2 and Oct4 in reprogramming. Altogether, these important studies in ES cells point to an important role of sequence-specific TFs in establishing which chromatin domains should remain accessible (in a hierarchical manner) after mitosis, in order to promote cell identity maintenance or cell fate changes. Curiously, it was recently shown that super enhancers regain accessibility after replication in ES cells faster than other genomic regions, through transcription mediated by TF occupancy [77,79], altogether suggesting the existence of a hierarchy of regulatory regions, whenever transcription is reactivated.

A recent study in *Drosophila* has shown retention of the neurogenic TF Prox1 on pericentromeric regions of mitotic chromosomes in dividing ganglion mother cells (GMCs), to be required for their terminal differentiation into post-mitotic neurons [80]. Mitotic retention of Prox1 results in the recruitment and concentration of the heterochromatin-forming protein HP1 into LLPS condensates, promoting H3K9me3 expansion in newly born neurons. Inclusion in LLPS condensates can be seen as a strategy to increase protein concentration in the vicinity of chromatin. Future studies should address whether this mechanism is of more general use by mitotic chromosome binders, possibly via IDRs known to promote such condensates [3,80]. Strikingly, the mitotic retention of Prox1 does not occur in the asymmetrically dividing neuroblast (NB) giving rise to GMCs. The *de novo* interaction of Prox1 with mitotic chromosomes in GMCs (which the authors refer to as ‘mitotic implantation’) can be explained by alterations in the cellular distribution of Prox1 (found tethered to the cell membrane in NBs), and does not necessarily entail changes to its ability to associate with mitotic chromosomes. Nevertheless, this is an important first example of dynamic regulation of MCB by a TF, in the context of a cell fate transition.

**Figure 3.**
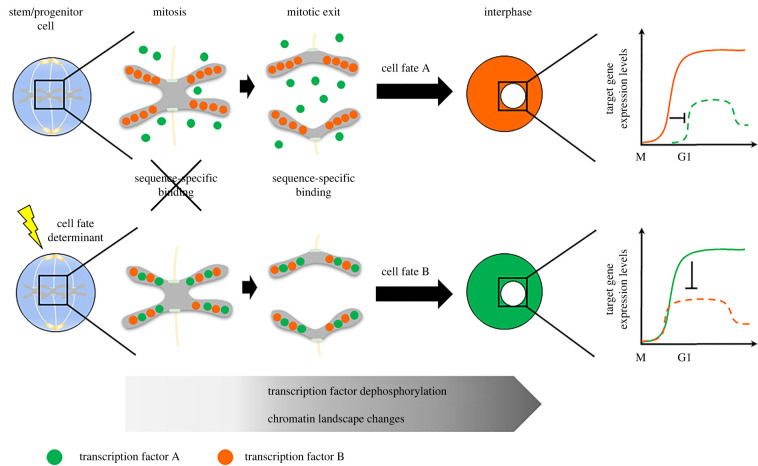
A model integrating dynamic changes in MCB with cell fate decisions. In the absence of sequence-specific binding, MCB may still contribute to an early time of transcriptional reactivation, by increasing TF concentration in the vicinity of chromatin when transcription is being restarted. Alternative cell-identity programmes are often antagonistic in nature. Thus, an early time of transcriptional reactivation may result in an advantage over the alternative cell-identity programme, providing a mechanistic link between MCB and cell fate. We propose that an extrinsic regulation of MCB can play a role in the execution of cell-fate decisions by cell-fate determinants (see main text for references).

How can modulation of MCB and changes in the temporal pattern of gene reactivation impact the activity of TFs with a role in cell fate decisions? The mutually exclusive nature of alternative cell fates results often from antagonistic cross-regulatory interactions between their associated transcriptional programmes. Thus, the ability of one of such TFs to reactivate its target genes early during M-G1, may confer a significant advantage over others. In this context, one interesting possibility is that signalling pathways regulating cell fate decisions may operate at one level by governing the association of TFs with mitotic chromosomes, and consequently the timing of reactivation of their programmes (figure 3). Investigating this model will require the capacity to survey the interaction of TFs with roles in cell-identity, with chromatin during M-G1, and how this relates to the fate of daughter cells. This is a major question in this exciting field that must be urgently addressed in future studies.

## Data Availability

This article has no additional data.
